# Child and Adolescent Mental Health Services in South Africa—Senior Stakeholder Perceptions of Strengths, Weaknesses, Opportunities, and Threats in the Western Cape Province

**DOI:** 10.3389/fpsyt.2019.00841

**Published:** 2019-11-26

**Authors:** Stella Mokitimi, Kim Jonas, Marguerite Schneider, Petrus J. de Vries

**Affiliations:** ^1^Division of Child and Adolescent Psychiatry, University of Cape Town, Cape Town, South Africa; ^2^Red Cross War Memorial Children’s Hospital, Division of Child and Adolescent Psychiatry, University of Cape Town, Cape Town, South Africa; ^3^Adolescent Health Research Unit (AHRU), Division of Child and Adolescent Psychiatry, University of Cape Town, Cape Town, South Africa; ^4^Health Systems Research Unit, South African Medical Research Council (SAMRC), Cape Town, South Africa; ^5^Alan J Flisher Centre for Public Mental Health, Department of Psychiatry and Mental Health, University of Cape Town, Cape Town, South Africa

**Keywords:** child, adolescent, mental health services, health systems, tipping point, South Africa, Africa, low- and middle-income countries

## Abstract

**Background:** There is general consensus that child and adolescent mental health services in low- and middle-income countries have an urgent need to be strengthened. However, this require not only a universal understanding of services and service needs, but also in-depth local knowledge to inform relevant service strengthening. This study sought to explore the perspectives of senior child and adolescent mental health service providers and policy-makers in one South African province to identify strengths, weaknesses, opportunities, and threats to child and adolescent mental health services.

**Methods:** A qualitative study was conducted with 13 purposively sampled senior child and adolescent mental health service providers, senior managers, and policy-makers from the Western Cape Province, using a half-day multi-stakeholder workshop format. Verbal and written data were recorded and coded for analysis. Two independent raters performed thematic analysis.

**Results:** The comprehensive bio-psycho-social approach and strong specialist child and adolescent mental health service units were identified as strengths. Limited capacity, workload demands, inadequate and inequitable resource allocation, poor implementation of early detection and preventative policies, and overall neglect of child and adolescent mental health services, were identified as weaknesses. Collaborative working between child and adolescent mental health and pediatric services, and increased provincial government (Department of Health) involvement, were identified as potential opportunities to develop and strengthen child and adolescent mental health services. Silo working of agencies, societal stressors, inadequate infrastructure and other resources, and lack of dedicated funding for child and adolescent mental health, were identified as threats to the development of services.

**Conclusions:** This analysis of strengths, weaknesses, opportunities, and threats reinforced the widespread neglect of child and adolescent mental health services in South Africa and highlighted areas for further research and advocacy. There is a clear need to explore the perspectives and experiences of service users and providers to generate comprehensive multi-stakeholder evidence that may identify positive "tipping points" for improvements and strengthening of child and adolescent mental health service delivery, training, and research.

## Introduction

Mental health disorders are the number one leading burden of disease in children and adolescents (1) affecting 10–20% of children and adolescents around the world. Strikingly, 50–80% of all adult mental health disorders emerge before the age of 18 (2). Mental health disorders in children and adolescents have a negative impact on their development and wellbeing. Children with mental health disorders often experience challenges in education and learning, in their transition to adult life, and in their potential to live fulfilling and productive lives (3). Child and adolescent mental health (CAMH) is therefore increasingly recognized as a public health priority (4–7). In spite the fact that 90% of the world's children and adolescents live in low- and middle-income countries (LMICs), the evidence-base for the burden of child and adolescent mental disorders in LMICs is very limited (8–11) and suggests a clear lack of policy development and policy implementation, very limited research, and very limited resources for CAMH services (11, 12).

**Figure 1:**
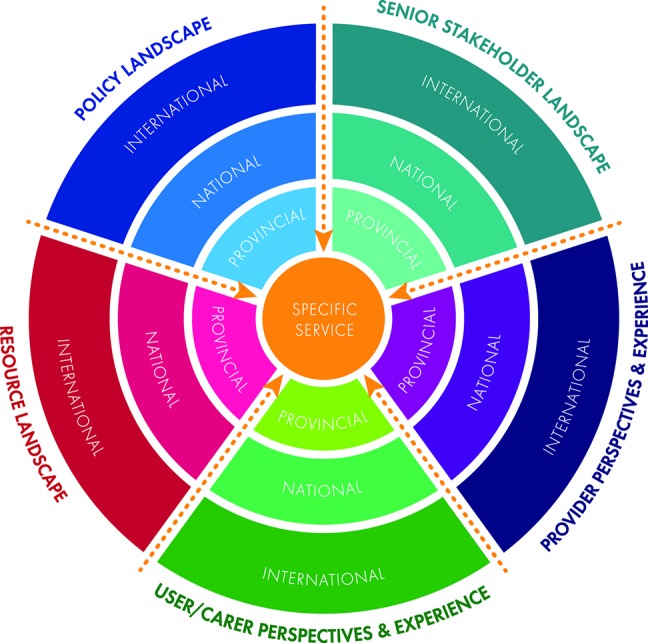
The multi-level integration of knowledge required to understand and strengthen child and adolescent mental health services.

Even though there is a clear global need for CAMH policy and service development, it is imperative that an understanding of these global needs is combined with local knowledge about health and care systems, existing resources, and local policies, particularly in LMICs. This requires a multi-level synthesis of available data including situational analysis of existing infrastructure, resources and workforce (13), evaluation of existing policies and policy implementation, and multi-level views of existing services and future service needs (13). A key component of local knowledge is therefore to have a thorough understanding of the perspectives of a broad range of stakeholders—from senior policy-makers and CAMH leadership, to clinicians who provide and families who receive services at the grassroots. **Figure 1** shows a graphic representation of the multiple levels that will require integration to understand and strengthen CAMH services. Levels include the "policy landscape" (international, national and regional/provincial knowledge, and perspectives on CAMH-relevant policies), the "resource landscape" (international, national, and regional/provincial knowledge about available infrastructure, human resources, and funding), the "senior stakeholder landscape" (international, national, and regional/provincial knowledge and perspectives of decision-makers and senior leadership in CAMH), "provider perspectives and experience" (of those working at the grassroots of service delivery), and "user perspectives and experience" (of families and young people who seek clinical services in a particular setting). A careful understanding is required at all levels relevant to a specific service in order to know how to approach service strengthening. Such understanding can identify the strengths, weaknesses, opportunities, and threats (SWOT) affecting the provision of optimal CAMH services.

South Africa is classified as an upper-middle income country by the World Bank (14). Importantly, South Africa is also recognized as the country with the greatest income inequality in the world (13) and as a result, has some of the greatest health disparities in the world (15). The 2018 mid-year population estimates showed that the country had a total population of 57.7 million of which 21.8 million (37.8%) were estimated to be under the age of 19 (16).

The global prevalence rate of CAMH disorders is estimated to be between 10 and 20% (5, 7). There are no prevalence studies on the mental health of children and adolescents in any Sub-Saharan African country, including South Africa. However, in South Africa Kleintjes and colleagues (17) generated estimates of children likely to have diagnosable CAMH disorders, based on international data. They showed that 17% of children were likely to have a diagnosable CAMH disorder (17), with the most common being generalized anxiety disorder (11%), followed by posttraumatic stress disorder and major depressive disorder/dysthymia (both 8%), oppositional defiant disorder (6%), and attention deficit/hyperactivity disorder (5%). Of note, no estimates were made for autism spectrum disorder and other main categories of neurodevelopmental disorders.

The last situational analysis of South African CAMH services took place in 2005 (11) and showed that there was a national CAMH policy (18), but that none of the South African provinces had a specific CAMH policy or implementation plans based on the national policy. There was inadequate and inequitable distribution of CAMH resources with most services located in the metropolitan areas of the country, limited specialist human resources for CAMH services, and a lack of human resource training of generalist workers in CAMH (11). The impact of stigma, the low priority of mental health, and lack of attention to the link between poverty and mental ill-health were proposed as factors that influenced the lack of developments in CAMH services (11).

In a recent investigation, we reviewed all national and provincial mental health policies to establish the current "policy landscape" for CAMH in South Africa (19). In South Africa, policy is set at national level, and implementation is delegated to provincial level, in acknowledgement of the highly diverse socio-economic and socio-cultural diversity of the country. Apart from the 2003 national CAMH policy (18), no South African provinces had CAMH policies or implementation plans to support the national CAMH policy (19). The main focus of the provincial health policies was on HIV/AIDS, tuberculosis, and on maternal health and child mortality. Policy documents made little or no mention at all of CAMH services (19). Our findings therefore confirmed the ongoing neglect of CAMH at policy level, in spite of the burden of CAMH disorders.

**Figure 2:**
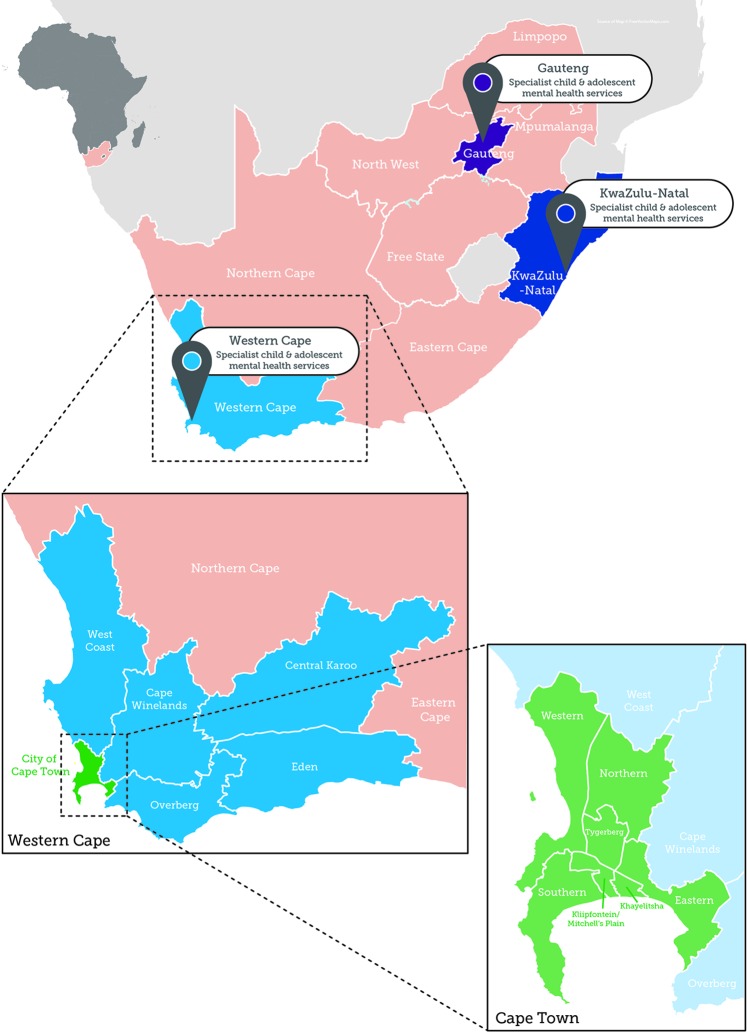
A map of South Africa indicating the location of specialist child and adolescent mental health services (indicated as pins on the map). The enlarged area shows the Western Cape Province and indicates the details of the different health districts and metropolitan sub-structures where the specialist CAMH services are situated.

As a next step toward an evidence-based, comprehensive CAMH service model in South Africa, it was therefore important to examine the "senior stakeholder landscape" (see **Figure 1**) by investigating local knowledge about CAMH services as perceived by senior and experienced policy makers and service providers. We elected to use the Western Cape Province as a case study for other low- and middle-income settings, given that it is the location of our clinical and academic activities. The Western Cape Province is one of nine provinces in South Africa, with Cape Town as the capital city. It has an overall population of 6.6 million (16), of which 2.2 million (33.3%) are under the age of 19 (16). The Western Cape and Gauteng Provinces are better resourced in terms of specialist CAMH services compared to other South African provinces (9). **Figure 2** shows a map of South Africa, indicating the location of provinces, and the location of specialist (tertiary) CAMH services. Specialist services are available in Gauteng (four service units), in Kwa-Zulu-Natal (one service unit), and in the Western Cape (three service units). There are no state/government-funded specialist CAMH services outside these centers.

In this study we therefore sought perspectives of senior CAMH service providers, managers, and policy-makers from the Western Cape Province of South Africa, to identify their multi-stakeholder perspectives of strengths, weaknesses, opportunities and threats (SWOT) in relation to CAMH services. Work by the Nobel Prize-winning economist Thomas Schelling in the 1970s (20) and popularized by Gladwell in 2000 (21), generated the concept of a "tipping point," defined as "the critical point in a situation, process or system beyond which a significant and often unstoppable effect or change takes place." The construct has been applied in various fields, including sociology and health systems research (22, 23). Gladwell (21) used the concept of tipping points to refer to potentially small events that could lead to significant change. We therefore set out to identify potential positive and negative "tipping points" that might be able to inform CAMH service strengthening activities from the data.

## Material and Methods

### Study Design

A qualitative study was conducted with purposively sampled senior CAMH service providers, senior managers, and policy-makers from the Western Cape Province, using a half-day multi-stakeholder workshop format.

### Study Participants

Purposive sampling was used to select relevant stakeholders with insight into CAMH issues to ensure representation across a wide range of experienced CAMH service providers and policy makers at all levels of care. We aimed to include policy-makers from the Western Cape Department of Health (DoH), senior CAMH managers, and senior practitioners from the three metropolitan sub-structures (Southern/Western sub-structure, Mitchell's Plain/Klipfontein sub-structure, Northern/Tygerberg sub-structure), from the rural districts (Cape Winelands, West Coast, Overberg, Eden and Central Karoo), and from all levels of care in the province (community, district, and tertiary care) were invited to participate. Details of the Western Cape, its rural districts and metropolitan sub-structures are shown in **Figure 2**.

A list of all mental health professionals and their contact details was obtained from the Provincial Mental Health Directory (24). The stakeholders were recruited telephonically and *via* email. A total of 24 multidisciplinary stakeholders were invited to participate.

### Setting and Data Collection

Information sheets containing details about the study and consent forms were emailed to participants prior to a face-to-face workshop held in March 2017. Willing participants who were not able to attend the meeting in person were provided with the key questions and were asked to return written comments for inclusion in data analysis. All consent forms were collected on the day of the workshop. The stakeholder engagement workshop was conducted in a quiet, private space at a central venue.

Participants were divided into three groups of four professionals each. Groups were structured to ensure a mix of professionals from the following categories: policy-makers, clinical psychologist, mental health nurses, nursing managers, child and adolescent psychiatrists, and medical officers (medically qualified professional without formal qualifications in psychiatry). Each small group was asked to perform a SWOT analysis in their group. Following small-group work, each group fed back to the large group, which led to additional large group discussion around similarities and differences identified. The lead author facilitated the large group discussions.

South Africa has 11 official languages. English is spoken as primary language by a small proportion of the population (∼10%), but it is the accepted language of communication at professional and senior stakeholder level. All participants were asked in advance of the workshop about their English proficiency, and all indicated that they were fluent in English. For this reason, all discussions were held in English. Discussions were audio-recorded and field notes were taken during the discussions and consolidated after the session. The duration of the stakeholder engagement workshop was three hours.

Four main aspects were discussed: strengths, weaknessess, opportunities and threats in CAMH services in the Western Cape. We conceptualized these as "current" positives (strengths) and negatives (weaknesses) *versus* "future" positives (opportunities) and negatives (threats). Member checking was done during the discussion process where the facilitator summarized the key points from the discussions and asked the participants to confirm these. Two of the authors (MS, PdV) were observers of the workshop but were not active participants in the discussions.

### Data Analysis

Audio recordings were analyzed using thematic analysis (25, 26) through NVIVO 11 (QSR) qualitative data software package (27). Relevant sections were transcribed verbatim in NVIVO. Data were coded, and codes were subsequently grouped into themes. The coded transcripts were analyzed by running query reports, and primary document tables were produced of the codes and themes to explore the issues from the discussions.

To strengthen the trustworthiness of analysis data triangulation was performed. Written notes from the workshop were made by the research team and these were compared with the data written by group participants, and the audio recordings from the workshop and corroborated the findings of this study. Furthermore, to ensure objectivity of the data two researchers (SM and KJ) coded all the data independently. The two researchers met regularly to compare and discuss their findings until consensus and saturation was reached. For this reason, no quantitative measure of agreement was calculated.

### Informed Consent and Ethics Approval

This study was approved by the University of Cape Town Human Research Ethics Committee (HREC 188/2016) and permission to conduct the study was received from the Western Cape DoH and the Red Cross War Memorial Children's Hospital. The study adhered to the principles as set out in the Helsinki Declaration (28). All participants were provided with a participation information sheet, and all provided written, informed consent before participating.

## Results

A total of 13 multidisciplinary stakeholders (12 face-to-face and 1 written response) participated in this study. Eleven invited professionals did not participate. The reasons for non-participation were stated to be time constraints and occupational requirements. Participants included one policy-maker, four advanced senior mental health nurses, two nursing managers, three child and adolescent psychiatrists (consultants), one medical officer (medically qualified professional without formal qualifications in psychiatry), one general psychiatrist, and one senior clinical psychologist. All the participants were experienced CAMH service providers. The years of CAMH service experience ranged from 4 to 20 years. The results of the multi-stakeholder engagement workshop are presented below according to the SWOT identified in thematic analysis.

### Strengths

Two main strengths emerged from the analysis. These were recognition of a comprehensive bio-psycho-social approach, and strong specialist CAMH service units. **Table 1** provides a summary of the identified themes and subthemes illustrated with representative quotes from participant transcripts.

**Table 1:** A summary of the themes illustrating the strengths of child and adolescent mental health (CAMH) services.

Overarching theme	Sub-themes	Illustrating examples/quotes
Comprehensive bio-psycho-social approach	Holistic across ages	* “combined child and adolescents … holistic across ages … excellent tertiary services”* ***[Child and adolescent psychiatrist]***
	More than custodial care	*“…we have a good vision for how CAMH services should exist, that it's more than custodial care…”* ***[Child and adolescent psychiatrist]***
Strong specialist CAMH service units	Clearer referral care pathways within CAMH services	“The referral care pathways are clearer now … like the person staying in Crossroads* cannot just refer a child to the Child and Family Unit …” ***[Child and adolescent psychiatrist]***
	Links between tertiary and primary CAMH services.	*“Training is provided for community Mental Health providers by the tertiary CAMH specialists…”* ***[Child and adolescent psychiatrist]*** *“Weekly supervision is provided for community Mental Health nurses on Fridays once a month…”* ***[Child and adolescent psychiatrist]***
	Improved training for primary CAMH services	*“There are joint CAMH case discussions at least once a term across the platform…”* ***[Child and adolescent psychiatrist]***
	Committed CAMH specialists	*“…people like us … who still care about it and … I mean there are people … committed … committed workforce. I mean nobody does this for fun…”* ***[Child and adolescent psychiatrist]***
	Highly skilled tertiary CAMH services	*“…There's some highly qualified multidisciplinary teams at all level …”* ***[Child and adolescent psychiatrist]***
		*“We’ve got skilled and experienced tertiary layer…”* ***[Child and adolescent psychiatrist]***
	Strong academic support and involvement for CAMH services	*“academic support … I mean they [university] do not just teach, but they do”* ***[Child and adolescent psychiatrist]***

*Crossroads: denotes a high-density residential area in Cape Town.

#### Comprehensive Bio-Psycho-Social Approach

CAMH services were described as having a "comprehensive bio-psycho-social approach," meaning the generation of a thorough formulation and intervention plan based on a comprehensive evaluation of the biological, psychological, and social needs of a child and their family. CAMH services were seen as "holistic" because they included preventive, promotive, and curative elements, and were offered at all levels of care—primary, secondary, and tertiary levels. The services were provided for ages 0–18 years, and assessment and interventions were described as developmentally appropriate to the needs of the child.

#### Strong Specialist Child and Adolescent Mental Health Service Units

The existing specialist units were described as understanding that CAMH services do not only comprise of custodial care but involve a holistic approach. The specialist units were said to offer in-patient and outpatient services and a range of psychotherapeutic interventions. Senior stakeholders reported that CAMH services had evolved a lot since 2014. For 3 years (2014 to 2017) CAMH services consistently had skilled and experienced clinicians. The specialists were described as passionate about what they do, striving to provide effective CAMH services despite the challenges. These CAMH specialists were described as having a vision of how services should be, and this vision was reportedly shared and implemented very effectively in various forums that meet on a regular basis. These meetings include highly qualified multidisciplinary team members from all levels of care. Participants described that information about CAMH services and referral care pathways were shared across the districts and across disciplines, thus raising awareness about CAMH services and referral pathways. The goodwill from the district level was recognized as a facilitator to sharing of best practices and increased awareness of CAMH services.

Participants commented that the Western Cape Province was advantaged compared to other provinces in the country with three strong and well-structured tertiary CAMH units: Lentegeur Child and Family Unit (linked to Stellenbosch University), the Tygerberg Child and Adolescent Psychiatry Unit (linked to Stellenbosch University), and the Division of Child and Adolescent Psychiatry at Red Cross War Memorial Children's Hospital (linked to the University of Cape Town). The three tertiary specialist CAMH units were described as the strengths and pillars of CAMH services in the Province. They were also perceived by the participants as the strongest CAMH units in the country with a reputation for providing excellent tertiary CAMH services that are comprehensive across ages.

CAMH services were receiving some support from the Western Cape Government DoH which included interactions and discussions with CAMH specialists about CAMH services. There were also interactions between CAMH specialists and district managers about CAMH services, and in some districts, multidisciplinary health teams had been formed that included a CAMH specialist. CAMH services also received support from the academic systems (University of Cape Town and Stellenbosch University) through training, clinical supervision, and research.

Tertiary CAMH specialists offered support to mental health providers and non-specialists in primary and secondary care levels through supervision and training. As a result of these interactions, referral care pathways had been improved in recent years.

### Weaknesses

Five main weaknesses emerged from the analysis—limited capacity, workload demands, inadequate and inequitable resource allocation, poor implementation of early detection and preventive policies, and overall neglect of CAMH services. **Table 2** provides a summary of the identified themes and subthemes illustrated with representative quotes from participant transcripts.

**Table 2:** A summary of themes illustrating the weaknesses in child and adolescent mental health (CAMH) services.

Overarching theme	Sub-theme	Illustrative example/quote
Limited capacity	Insufficient training in CAMH services	*"…there's insufficient focused training on child and adolescent psychiatry…"* ***[Nursing manager]***
Workload demands	Workload measurement not correlating to child psychiatry	"Stats* is a problem … if my output is about 25 patients per month…that doesn't speak to my workload … doesn't speak to how many crises I've had in between…" ***[Advanced senior psychiatric nurse]***
Inadequate and inequitable resource allocation	Unequal distribution of CAMH services	*"…we're dealing with a large gap of socioeconomic status … so there's a large variation of accessibility for services and knowledge about the services etc…"* ***[Child and adolescent psychiatrist]***
	Dependency on NGOs**	"I think that too many things are left to NGOs**…" ***[Advanced senior psychiatric nurse]*** *"Too much is now left to NGOs where they are now seeing these children…"* ***[Advanced senior psychiatric nurse]***
Poor implementation of early detection and preventive policies	Preventative approaches not implemented	*"…preventative approaches … that's not being implemented…"* ***[Advanced senior psychiatric nurse]*** *"There's no early detection and prevention for CAMH services … we only get them once it's a train smash and there's a lot of services that we need…"* ***[Child and adolescent psychiatrist]***
Overall neglect of CAMH services	Poor knowledge of the needs of CAMH services	*"There's lack of understanding of child and adolescent mental health, it's still termed naughtiness even with educational services…"* ***[Advanced senior psychiatric nurse]*** *"…it's very difficult to put a voice to those people who don't know what we do, versus what they do … they don't understand what the need is this side…"* ***[Nursing manager]***
	Lack of priority for CAMH services	*…a psychiatric emergency versus medical emergency … there's not so much recognition … like a patient in ICU…"* ***[Advanced senior psychiatric nurse]*** *"They don't see a psychiatric emergency like a medical emergency … less urgent…"* ***[Advanced senior psychiatric nurse]*** *"A psychiatry emergency is always less important than a medical emergency…"* ***[Advanced senior psychiatric nurse]***
	Low levels of advocacy for CAMH services	*"…we haven't got the bodies to do the advocacy…"* ***[Child and adolescent psychiatrist]*** "In the actual fact there's lots of advocacy but it's been busy with Esidimeni…"*** ***[Child and adolescent psychiatrist]***

*A local term used to refer to the workload expectation in services i.e., the requirement to see a certain number of patients per day in a service.

**Non-governmental organizations.

***Referring to the Life Esidimeni Crisis (2018) when 118 adults with mental health disorders and/or intellectual disability died after transfer from specialist facilities to non-registered NGOs.

#### Limited Capacity

Participants described a generally lack of capacity in the Western Cape within the DoH, the Department of Education (WCED), and the Department of Social Development (DSD). The WCED and DSD were described as having high workloads and a shortage of resources to meet the demand. DoH staff felt that WCED and DSD referred a lot of inappropriate cases to CAMH services, adding to the workload for the DoH. Participants referred to insufficient training on CAMH within all three departments, and expressed concern about lack of standardized best practices across CAMH services. Limited human resources within the DoH for CAMH services were described, particularly at primary and secondary levels of care. Non-specialists were described as being overwhelmed by the CAMH workload, despite their goodwill.

#### Workload Demands

There were complaints about the "stats" requirements for staff in CAMH services. In this context, "stats" is a local term used to refer to the workload expectation in services, in other words the requirement to see a certain number of patients per day in a service. Service providers felt that these "stats" requirements did not correlate with the work that they do. The pressure on service providers to meet the "stats" quotas set by senior managers was seen to be at the expense of the quality of service that was needed by users. As a result, clinical staff felt as if they were not doing enough when low numbers were reflected. Service providers felt strongly that the type of "stats" quotas for CAMH services should reflect and capture all the qualitative therapeutic work done and not just be about numbers. Participants reported that generalists in primary health care settings and secondary level were also unable to manage their "stats" demands given the high workload associated with CAMH cases.

#### Inadequate and Inequitable Resource Allocation

Participants reported that there were no outpatient, inpatient, or inpatient emergency facilities for CAMH services at secondary level (that is, at district/regional hospital level). Children were still mixed with adults at primary and secondary levels of care. CAMH resources were still unequally distributed in the Western Cape thus limiting access to CAMH services for those who do not live in Cape Town close to specialist facilities. Participants further commented that there were fewer resources and access to CAMH services for areas with the lowest socioeconomic status, including rural areas.

Non-governmental/non-profit organizations (NGO/NPOs) and academic institutions were seen as providing good support to the overburdened DoH CAMH services. Academic institutions were recognized not only as conducting research and teaching, but also as providers of clinical services to children and their families. NGO/NPOs were recognized as providing services for a lot of cases that could not be seen in the DoH CAMH services. However, participants expressed the view that the NGO/NPO sector was "overused" as a way of compensating for the lack of government-funded CAMH services.

#### Poor Implementation of Early Detection and Preventive Policies

Despite the reported strength of the presence of preventive service plans, concern was expressed that these policies and plans had not been implemented. Early detection of CAMH problems was lacking, cases were often described as being referred only when there is a crisis or when the problem has worsened and become complicated by which time complex, long-term, and multiple resources are typically required for intervention. Participants reported that the preventive work becomes the burden of the parents and families who are expected to take initiative to ensure care for their children.

#### Overall Neglect of Child and Adolescent Mental Health Services

Participants reported that the needs for CAMH services were not prioritized and often not met in comparison with other medical disciplines, particularly for emergencies. Given that CAMH services are dependent on budget allocation within facilities, CAMH specialists in those facilities often have to advocate for the needs of CAMH services. However, these attempts were described as "often in vain" due to limited insight of those in managerial positions, of non-specialists, and those who allocate budgets. Participants expressed concern about a lack of insight and understanding about what CAMH services entail and about exact needs of CAMH services, in spite of ongoing information, advocacy, and education within facilities and institutions. Participants acknowledged that advocacy for CAMH services at a high-level provincial and national level was actually lacking and that existing advocacy in mental health had mainly focused on the "Life Esidimeni" crisis (29) which solely focused on the mental health needs of adults with and without intellectual disability. The "Life Esidimeni" crisis involved the death of 118 adults with mental health problems and/or intellectual disability in 2018 when they were forcibly removed from "Life Esidimeni" psychiatric homes and placed in ill-equipped, unprepared, and unlicensed NGOs.

### Opportunities

The following were identified by participants as opportunities for development and strengthening of CAMH services in the Western Cape: collaborative working between CAMH and pediatric services, and increased provincial Government (DoH) involvement. **Table 3** provides a summary of the identified themes and subthemes illustrated with representative quotes from participant transcripts.

**Table 3:** A summary of themes illustrating the opportunities to improve child and adolescent mental health services.

Overaching theme	Sub-themes	Illustrative examples/quotes
Collaborative working between CAMH services and pediatric services	Early identification of CAMH problems	*"There was a proposed merger of pediatricians and child psychiatrists. The first 1000 days … the pediatricians are reporting on the first 1000 days … actually the first 1000 days is a facilitator…"* ***[Child and adolescent psychiatrist]***
Increased Provincial Government (Department of Health) involvement		*"There's now some support from the Department [Department of Health] for the last three years…"* ***[Child and adolescent psychiatrist]***

#### Collaborative Working Between Child and Adolescent Mental Health and Pediatric Services

The "first 1,000 days" project, a collaborative cross-agency program involving the DoH, WCED, and DSD (30), was created as a systematic preventative program to identify and reduce risk factors for maternal and infant mental health problems in the first 1,000 days of life. The program was given as example of an approach that helped to establish and build relationships across agencies (DoH, WCED, and DSD) and between different disciplines, and led to reporting on health indicators in the first 1,000 days of life. It was seen as instrumental in creating awareness among pediatricians about maternal and infant mental health, which improved interaction and cross-referrals between CAMH and pediatrics services. The project was therefore identified as a model to improve integrated services across agencies and disciplines working with children and adolescents at risk of mental health problems.

#### Increased Provincial Government (Department of Health) Involvement

Participants described that there had been increased interaction between the Provincial DoH and CAMH specialists over a 3-year period (2014–2017), with an interest from the Provincial DoH to understand CAMH services and service needs. This was seen as an opportunity for CAMH specialists to advocate for the needs of CAMH services.

### Threats

The following threats for CAMH services in the province were identified: Silo working of agencies, societal stressors, inadequate infrastructure and other resources, and lack of dedicated funding for CAMH services. **Table 4** provides a summary of the identified themes and subthemes illustrated with representative quotes from participant transcripts.

**Table 4:** Provides a summary of themes illustrating the threats for child and adolescent mental health (CAMH) services.

Overaching theme	Sub-themes	Illustrative examples
Silo working of agencies	Lack of multisectoral collaboration	*"The department of social development and the Western Cape education are the two biggest headaches … if they don't know which way [to refer patients] it becomes health's problem, and inevitably because it's not physical health it ends up in mental health … they are overburdened … they are flooded…"* ***[Advanced senior psychiatric nurse]***
		*"lack of multiagency joint working. We're very much dependent on … social workers and the department of education and when those aren't functioning, that impacts on our work…"* ***[Senior clinical psychologist]***
Societal stressors	Societal decay	*"Societal decay is affecting us [CAMH services] …"* ***[Advanced senior psychiatric nurse]*** *"Lack of structure … fractured families … lack of stability…"* ***[Advanced senior psychiatric nurse]***
	Fractured families	*"…We come from a system which has been traumatised over generation with both systematic violence like group segregation … migrant labour … the political situation in the country has facilitated the breakup of families … so the parenting has been done by grandparents … which were under-resourced … it was just the system that was trying to produce the generation with difficulties … we're sitting with a generational legacy which has not been addressed … trying to address the child's problem in the context of weak parenthood…"* ***[Child and adolescent psychiatrist]***
	Stigma	*"With children there's always stigma. They cannot defend themselves. That can be a barrier…"* ***[Child and adolescent psychiatrist]***
Inadequate infrastructure and other resources	Limited dedicated CAMH therapeutic facilities	*"The children now are being lost … there are a lot of children with psychosis … we've lost therapeutic services for children…"* ***[Policymaker]*** "There are no emergency psychiatric beds for children in this province, when TLC is full…"* ***[Advanced senior psychiatric nurse]*** *"There's no inpatient facilities for non-psychotic children…"* ***[Child and adolescent psychiatrist]***
Lack of dedicated finding for CAMH services	No separate funding for CAMH services	"we have a competition with more sexy … you know once we lost out to the penis transplant**…it's always about ICU***, and the neurosurgeries and the and the penis transplants and all the other stuff…" ***[Child and adolescent psychiatrist]*** *"CAMH services always have to compete with other departments for funding…"* ***[Child and adolescent psychiatrist]*** *"There is no separate funding for CAMH services…"* ***[Policymaker]***

*Therapeutic Learning Centre, a small inpatient unit for children with complex mental health problems.

**Innovative surgery performed for the first time in South Africa.

***Intensive care unit for physically ill children.

#### Silo Working of Agencies

Participants described a lack of "joined-up" or coordinated multi-agency work between the DoH, WCED, and DSD. All these departments were described as work in "silos," which made it difficult to manage cases that required intervention or input from all three agencies. There was a strong feeling that the challenges and service pressures within WCED and DSD impacted directly on the DoH, leading to inappropriate referrals to the DoH and struggles to do joint working across agencies.

#### Societal Stressors

Participants cited the high rates of poverty, crime, substance abuse, and violence in communities as resulting in psychiatric morbidity in children and adolescents. This was perceived to lead to a "revolving door" system for children and adolescents affected with mental health problems. Stakeholders described that treating mental health problems effectively when children live in maladaptive contexts and unsupportive communities becomes difficult to sustain. Many children were described as coming from traumatized backgrounds and fractured family structures. Parents often have mental health problems and intervention is needed for both the parent and the child. Senior stakeholders also reported significant stigma attached to child and adolescent mental illness and an associated lack of insight into CAMH problems. Psychiatric problems in children were often viewed as a child just "being naughty" or as parents not being able to discipline their child. Families who seek help from CAMH services were often stigmatized within their extended families, in their communities, and within the healthcare system.

#### Inadequate Infrastructure and Other Resources

Participants reported that in the whole of the Western Cape there were only three CAMH specialist units providing inpatient and outpatient services exclusively for children and adolescents. These units provide tertiary services and are therefore based only at the two tertiary teaching hospitals in Cape Town. The limited services and infrastructure was described not only as a weakness as described above, but also as a threat to future services and service delivery.

Participants expressed concern that there were no dedicated CAMH services at primary level (public health services at community level). Children and adolescents are seen together with adult psychiatric patients in outpatient psychiatric services that are not child/adolescent-friendly. Children and adolescents may therefore be traumatized by the aggressive or high-risk behaviors of adult patients with serious mental illnesses. At day hospitals psychotropic medications are inconsistently available (e.g., available for a few weeks, and then not available for the next month). This may risk worsening of the mental states of children and adolescents and/or development of treatment resistance. As outlined earlier, resources were most limited in the most needy and vulnerable communities such as in very low socio-economic or rural settings.

At secondary (district/regional) level, there are no dedicated facilities for CAMH problems—neither for outpatient care nor for psychiatric emergencies. Acute cases of children under the age of 12 years therefore have to be admitted to general pediatric wards, and adolescents over 13 years of age have to be admitted to adult psychiatric emergency inpatient units. These inpatient units are not child/adolescent-friendly and do not have the appropriate resources to assess children and adolescents. Often there are no therapeutic resources, such as developmentally appropriate reading or self-help guides or play materials to engage children and adolescents while in the unit. Units are also not designed to provide safety and privacy to a child/adolescent with an acute psychiatric problem. Service providers are not trained to manage the challenging behavior of the acutely mentally ill child or adolescent. Even more pronounced than in outpatient settings, adolescents are frequency exposed to aggressive and high-risk behaviors of adults with acute severe mental illnesses.

#### Lack of Dedicated Funding for Child and Adolescent Mental Health Services

There were no dedicated budgets for CAMH services at National or Provincial level. Participants reported that, at some stage in the past, provincial budgets were divided into mental health and general health budget and the mental health budget was dedicated and ring-fenced. The ring-fenced mental health budget was, however, discontinued and only mental hospitals now have dedicated budgets. CAMH is a predominantly outpatient-based service and there are no dedicated CAMH hospitals. The budget for CAMH services are therefore integrated within the general health budget within the facilities where CAMH teams are based, for instance at Tygerberg, Lentegeur, or Red Cross War Memorial Children's Hospital. Participants expressed the view that there was high competition with other departments and emergencies for budget in these facilities, and that CAMH services were often the least valued and prioritized, making it difficult to maintain essential resources or to improve the existing resources.

## Discussion

Noting the lack of improvement in CAMH services in South Africa over the years since the last situational analysis in 2005 (11), and the lack of progress in policy development and implementation (11, 19), we sought to obtain senior stakeholder perspectives on the current state of CAMH services to provide key local knowledge that could inform policy and service strengthening for CAMH in a South African context. The study therefore collected multistakeholder data from CAMH policy-makers, senior service managers, and senior service providers on the SWOT of CAMH services in the Western Cape Province of South Africa.

Stakeholders identified a number of positive aspects of CAMH services. These included recent improvements in services, strong specialist services at tertiary level, improved collaborative working with the provincial DoH government, and enhanced interaction between the specialist multidisciplinary teams and non-specialist colleagues at district and community level. These findings suggest potential strategies for CAMH service strengthening through ongoing collaboration with policymakers and funders, and ongoing training and capacity-building with district and community-level colleagues. Results may suggest that particular benefits may come from the identification of appropriate task-sharing activities in keeping with WHO recommendations (31).

The stakeholder findings also identified a significant number of negative aspects of CAMH services, many of which were similar to observations made more than 10 years earlier (11). Poor intersectorial working, limited and inappropriate resources, unreasonable expectations of CAMH staff, and absence of dedicated budgets for CAMH, all in the context of societal stressors, were seen as major barriers to CAMH services. Current proposals on budget allocation, the structuring of CAMH services and allocation of resources for CAMH services are therefore likely to threaten development of comprehensive CAMH services and compromise the efficacy of CAMH providers at all levels of care very significantly over the next decade. This, in turn, may result in greater costs to treat complicated CAMH problems in adulthood (12, 32).

**Figure 3:**
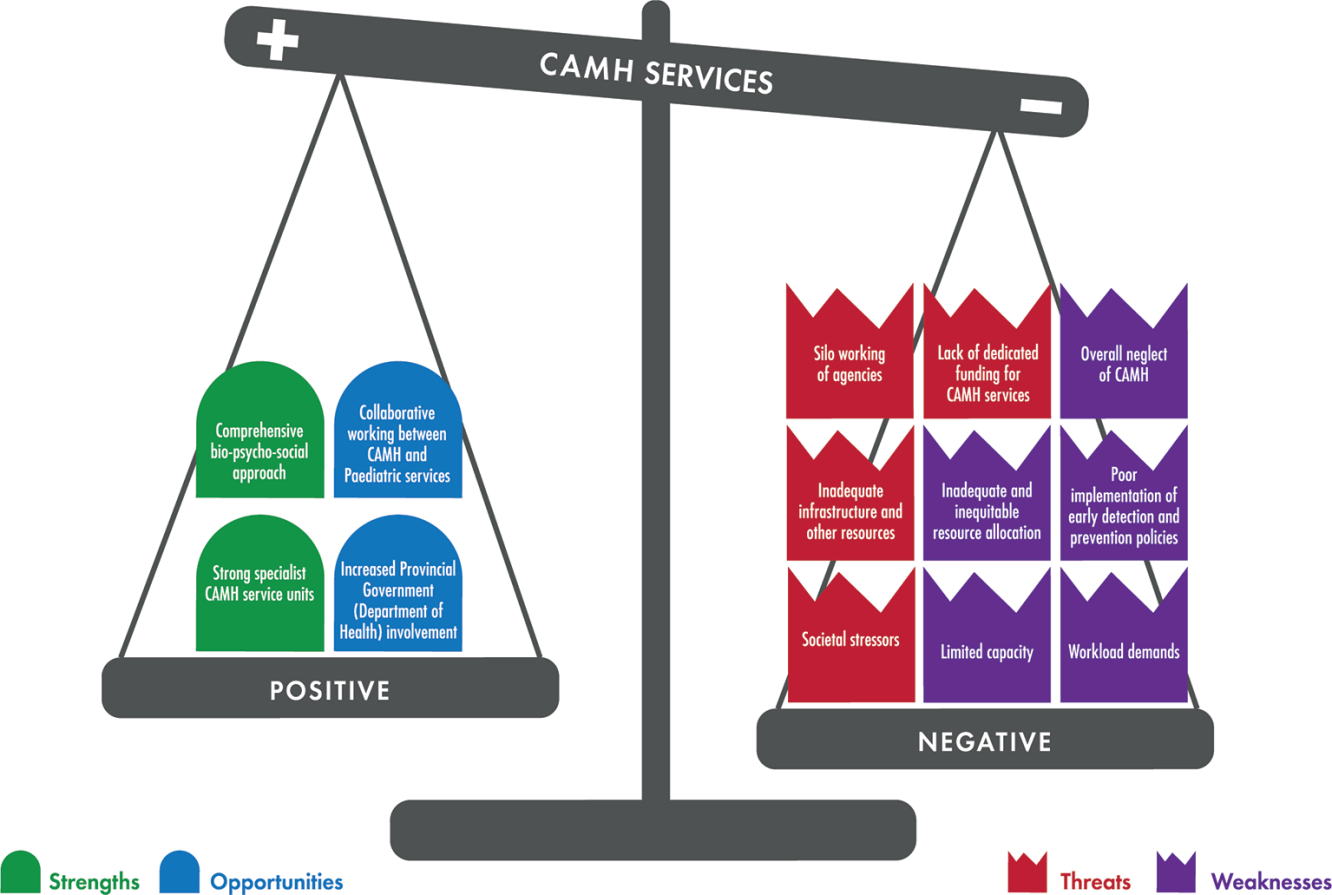
The tipping point model of child and adolescent mental health services.

In an attempt to generate a synthesis of the findings from this SWOT analysis, we sought a visual model that could help to integrate the otherwise potentially unrelated positive aspects outlined (strengths and opportunities) in relation to the negative aspects outlined (weaknesses and threats). Using the concept of "tipping points" in the context of our study, we propose that the strengths and opportunities *versus* the weaknesses and threats may contribute to a scale of potential for strengthening (or weakening) of CAMH services. **Figure 3** provides a graphic representation of this concept, incorporating the findings from this study. Data generated in this study suggests that, even though a number of positive elements were identified, a much larger number of negative elements of CAMH services may be present, threatening a tipping point toward disruption and weakening of services.

While these findings are of significant concern, particularly in a country which is often thought of as well-developed in terms of mental health and health services, the "tipping point model" of CAMH service strengthening may also allow us to identify small events, actions, and activities that could lead to significant positive change. From the data presented here, it seems that, for instance, the "first 1,000 days of life" project (which was a multi-sectorial project to identify and manage risks to mothers and their infants), and recent collaborative work between CAMH teams and Provincial DoH (to identify and strengthen some elements of CAMH services) may represent examples of positive tipping events. These can facilitate recognition of CAMH as a health priority and provide an opportunity for changing budget allocations to create parity with other medical problem. Such interactions may also influence policies that could formally define interactions, roles, and responsibilities between the four Government Departments directly involved in children and adolescents (DoH, WCED, DSD, and Justice).

It is clear that there are many unanswered questions about CAMH in the Western Cape and in South Africa that could shed light on potential positive tipping events for CAMH strengthening. For instance, the perspectives and lived experience of clinicians at the very grassroots of district and community service delivery, and those of families and children who access CAMH services may identify additional positive or negative tipping factors. It would be of particular importance to seek broad representation across socio-economic, cultural, linguistic, rural/urban, religious, and age-based variables.

### Limitations of the Study

We acknowledge that the data generated in this multistakeholder analysis were derived from one small group of high-level clinical, managerial, and policy-making stakeholders in one South African province. Great care should therefore be taken in generalization of findings. However, given that this was a qualitative study, the data were sufficient to generate saturated themes, supporting the robustness of findings. At least some of the themes and subthemes that emerged may, therefore, resonate with the needs of other provinces and LMICs. We further acknowledge that the study performed qualitative analysis of data which may be open to bias. However, to increase trustworthiness and robustness of findings, we used two independent raters (one a CAMH specialist, the other a non-clinician) to generate themes and subthemes. Member-checking during the workshop further added to the trustworthiness of results. It will be important to perform triangulation of data with subsequent studies (e.g., provider or user perspectives) to generate the most comprehensive findings. All discussions were held in English, even though not all participants were primary English speakers. However, as outlined in the *Material and Methods*, English is very much the "lingua franca" in professional settings, and all participants indicated that they were fluent in English. We were therefore confident that the quality of data was not compromised as a result of conducting the workshop in English. It would be important for "grassroots" level analysis to interview participants in their primary language, such as isiXhosa or Afrikaans. The absence of other service providers and of children and families in this manuscript may appear to be a limitation. However, given the importance of the user/carer perspective and the provider perspective (as shown in **Figure 1**), we have opted to dedicate two separate sub-studies and separate manuscripts to the voices of families and children who use CAMH services, and to those who provide services at the grassroots.

### Relevance of Findings to Other Low- and Middle-Income Countries

As outlined under limitations, we acknowledge that themes and subthemes identified may not all be of direct relevance to other LMICs. In fact, it is important to consider that different countries, communities, and settings may have very different CAMH service models and healthcare systems. It would therefore be of utmost importance to perform similar SWOT analysis in different settings that may identify similar or different "tipping" events or factors that could be used for CAMH strengthening activities in those settings. We predict that there may be some universal themes, subthemes, and "tipping points" across LMICs such as lack of policy development, inadequate CAMH resources, poor intersectoral collaboration, unclear financing for CAMH services, and the potential integration of CAMH services into general health services (12, 33). However, these should all be subject to empirical investigation.

## Conclusions

Findings of this SWOT analysis provided insight into senior stakeholder perceptions about the current state of CAMH services in the Western Cape province of South Africa. The weaknesses and threats to CAMH services identified here were clearly of concern. We propose, as a next step, that further exploration of clinician and user perspectives from the grassroots of CAMH services should be investigated to identify positive "tipping" events, activities, or behaviours that could be incorporated into a comprehensive strategy to strengthen CAMH services in South Africa, and that may be of potential value in other LMICs.

## Data Availability Statement

Data generated in this study are available from the authors with appropriate permissions. 

## Ethics Statement

This study was carried out in accordance with the recommendations of the University of Cape Town and Faculty of Health Sciences Human Research Ethics Committee with written informed consent from all subjects. All subjects gave written informed consent in accordance with the Declaration of Helsinki. The protocol was approved by the University of Cape Town Faculty of Health Sciences Human Research Ethics Committee (HREC 188/2016). Information sheets containing details about the study and consent forms were emailed to participants prior to a face-to-face workshop held in March 2017. All consent forms were collected on the day of the workshop.

## Author Contributions

SM, PV, and MS participated in the conception and design of the study. SM performed data collection. SM and KJ performed the data analysis and interpretation of the data and prepared the first draft of the manuscript. PV and MS contributed to analysis, interpretation of results, and writing of the manuscript. All authors participated in the reviewing of the content for submission. All authors approved the final version of the manuscript.

## Funding

To SM: Research Funding—Department of Psychiatry, University of Cape Town; Spirit of 68 Scholarships—University of Cape Town; The Western Cape Department of Health; SAMRC Research Development Grant. To PV: National Research Foundation, University of Cape Town, Struengmann Fund.

## Conflict of Interest

The authors declare that the research was conducted in the absence of any commercial or financial relationships that could be construed as a potential conflict of interest.

## Abbreviations

MH, mental health; CAMH, child and adolescent mental health; CA, children and adolescents; DoH, Department of Health; LMIC, low- and middle-income countries; SWOT, strengths, weaknesses, opportunities, and threats.
